# 肺结节大小、深度对肺切除治疗≤2 cm早期肺癌手术方式的影响

**DOI:** 10.3779/j.issn.1009-3419.2024.101.08

**Published:** 2024-03-20

**Authors:** Zaibin TANG, Wenke GE, Dingye ZHOU, Zhicheng HE, Jing XU, Xianglong PAN, Liang CHEN, Weibing WU

**Affiliations:** 210000 南京，南京医科大学第一附属医院胸外科; Department of Thoracic Surgery, The First Affiliated Hospital of Nanjing Medical University, Nanjing 210000, China

**Keywords:** 肺肿瘤, 手术方式, 深度, 大小, 亚肺叶切除, 切缘, Lung neoplasms, Surgical approach, Depth, Size, Sublobar resection, Margin distance

## Abstract

**背景与目的:**

现有研究显示，在确保安全切缘的前提下，≤2 cm含磨玻璃成分的早期肺癌宜采用亚肺叶切除，但部分病例需行肺叶切除以保证切缘。本研究探讨≤2 cm早期肺癌的大小、深度对楔形、肺段和肺叶切除手术方式的影响，以及如何确保亚肺叶切除的安全切缘。

**方法:**

回顾性分析2022年接受肺切除手术治疗的≤2 cm含磨玻璃成分的早期肺癌病例385例，包括楔形、肺段和肺叶切除术三组。深度测量肺结节内缘至所属肺支气管开口最短距离（OA值）及结节内缘至胸膜距离（AB值）。行肺段及肺叶切除术者，进行三维CT支气管血管重建（three-dimensional computed tomography bronchography and angiography, 3D-CTBA），统计若行肺段切除术所需切除亚段数。统计楔形、肺段切除的切缘宽度和肺段切除所切除的亚段及数量。

**结果:**

在楔形、肺段和肺叶切除手术中，肺结节平均大小分别为（1.08±0.29）cm、（1.31±0.34）cm、（1.50±0.35）cm，结节的深度（AB值）分别为6.05（5.26, 6.85）cm、4.43（3.27, 5.43）cm和3.04（1.80, 4.18）cm，均呈现逐渐增大的趋势（P<0.001）。肺段切除获得的中位切缘宽度为2.50（1.50, 3.00）cm，显著大于楔形切除的1.50（1.15, 2.00）cm（P<0.001）。当楔形切除切缘<2 cm时，AB值>2 cm的病例占29.03%，高于切缘≥2 cm时AB值>2 cm的占比12.90%（P=0.019），以结节大小为切缘标准时，切缘/直径<1较切缘/直径≥1，AB值>2 cm病例的比例依旧更高（37.50% vs 17.39%, P=0.009）。肺段组切除的中位亚段数为3个，肺叶组病例若行肺段切除须切除的中位亚段数为5个（P<0.001）。

**结论:**

肺癌结节大小和深度综合影响肺切除术方式的选择，本研究首次证实越深越大的肺结节需要切除更大范围的肺组织才能获得安全切缘，肺结节内缘距离最近胸膜≤2 cm可能是楔形切除的理想指征。

## Abstract (en)

**Background and objective:**

Current studies suggest that for early-stage lung cancers with a component of ground-glass opacity measuring ≤2 cm, sublobar resection is suitable if it ensures adequate margins. However, lobectomy may be necessary for some cases to achieve this. The aim of this study was to explore the impact of size and depth on surgical techniques for wedge resection, segmentectomy, and lobectomy in early-stage lung cancer ≤2 cm, and to determine methods for ensuring a safe resection margin during sublobar resections.

**Methods:**

Clinical data from 385 patients with early-stage lung cancer ≤2 cm, who underwent lung resection in 2022, were subject to a retrospective analysis, covering three types of procedures: wedge resection, segmentectomy and lobectomy. The depth indicator as the OA value, which is the shortest distance from the inner edge of a pulmonary nodule to the opening of the corresponding bronchus, and the AB value, which is the distance from the inner edge of the nodule to the pleura, were measured. For cases undergoing lobectomy and segmentectomy, three-dimensional computed tomography bronchography and angiography (3D-CTBA) was performed to statistically determine the number of subsegments required for segmentectomy. The cutting margin width for wedge resection and segmentectomy was recorded, as well as the specific subsegments and their quantities removed during lung segmentectomy were documented.

**Results:**

In wedge resection, segmentectomy, and lobectomy, the sizes of pulmonary nodules were (1.08±0.29) cm, (1.31±0.34) cm and (1.50±0.35) cm, respectively, while the depth of the nodules (OA values) was 6.05 (5.26, 6.85) cm, 4.43 (3.27, 5.43) cm and 3.04 (1.80, 4.18) cm for each procedure, showing a progressive increasing trend (P<0.001). The median resection margin width obtained from segmentectomy was 2.50 (1.50, 3.00) cm, significantly greater than the 1.50 (1.15, 2.00) cm from wedge resection (P<0.001). In wedge resections, cases where AB value >2 cm demonstrated a higher proportion of cases with resection margins less than 2 cm compared to those with margins greater than 2 cm (29.03% vs 12.90%, P=0.019). When utilizing the size of the nodule as the criterion for resection margin, the instances with AB value >2 cm continued to show a higher proportion in the ratio of margin distance to tumor size less than 1 (37.50% vs 17.39%, P=0.009). The median number of subsegments for segmentectomy was three, whereas lobectomy cases requiring segmentectomy involved five subsegments (P<0.001).

**Conclusion:**

The selection of the surgical approach for lung resection is influenced by both the size and depth of pulmonary nodules. This study first confirms that larger portions of lung tissue must be removed for nodules that are deeper and larger to achieve a safe margin. A distance of ≤2 cm from the inner edge of the pulmonary nodule to the nearest pleura may be the ideal indication for performing wedge resection.

肺癌是我国发病率和死亡率最高的恶性肿瘤。高分辨率计算机断层扫描（high-resolution computed tomography, HRCT）在临床上的普及，使越来越多的早期肺癌被检出。JCOG0804/0802和CALGB140503等研究^[1⇓⇓-4]^认为：亚肺叶切除（楔形、肺段切除）可有效治疗直径不超过2 cm的外周早期肺癌。除了大小，肺结节的深度也是可否行肺段切除的重要因素，虽然指南^[5]^仅推荐外周的早期肺癌可行亚肺叶切除术，但一些研究^[6⇓-8]^表明位于肺实质中1/3的深部小肺癌也具有肺段切除的机会。确保安全切缘是亚肺叶切除获得良好预后的关键^[9]^，而有限度的肺段切除范围是获得肺功能保护的前提^[10,11]^。根据肺段的锥式解剖原理，更深更大的肺结节需要切除更大范围的肺组织才能获得安全切缘，但在真实世界中，是否有此规律尚未可知。本研究回顾性分析单中心数据，分析≤2 cm肺癌结节的大小、深度对楔形、肺段和肺叶切除手术方式的影响，以及如何确保亚肺叶切除的安全切缘。

## 1 资料与方法

### 1.1 研究对象

回顾性分析2022年1月至12月于南京医科大学第一附属医院胸外科单治疗组完成肺结节手术治疗的927例患者的临床资料。纳入标准：肺结节影像学病灶最大径≤2 cm含磨玻璃成分的肿瘤原发灶-淋巴结-转移（tumor-node-metastasis, TNM）分期为cN0M0的早期肺癌；患者肺功能和重要脏器功能满足肺叶切除术。排除标准：同一肺叶手术切除肺结节数量大于1枚；术前CT不能分辨四级支气管结构病例；右中肺结节；肺裂发育不全且跨叶结节。共计385例病例纳入分析，其中55例行肺叶切除术，175例行解剖性亚肺叶切除术，155例行楔形切除术。本研究经医院伦理委员会批准（批准号：2022-SR-760），所有患者均知情同意。

### 1.2 数据收集

收集记录患者围手术期病史资料。将纳入研究病例的术前胸部CT图像数据传输至“InferVision”软件中，进行支气管、血管及病灶的三维重建。使用“InferVision”软件中切缘球模拟功能，设置2 cm切缘模拟球。根据模拟球与静脉及段间平面、亚段间平面的关系，统计病灶若行解剖性肺切除时所需要切除的肺亚段及亚段数量。在二维测量中，将靶肺段开口作为O点，将肺段开口至结节中心的连线与结节内缘和壁层胸膜的交点作为A点和B点，使用RadiAnt DICOM Viewer软件中3D多平面重建功能分别测量肺段开口至胸膜的距离（OB）、肺段开口至结节内缘的距离（OA）及结节内缘至壁层胸膜的距离（AB）。手术切缘由病理科高年资医生测量记录，手术切缘宽度定义为从原发肿瘤到最近的钉线距离。所有患者术后第2周、第3个月，再后每6个月门诊随访。

### 1.3 数据分析

采用SPSS 26.0进行统计学分析。符合正态分布的计量资料采用均数±标准差表示，并用t检验或方差分析进行比较，不符合正态分布的计量资料采用中位数及四分位数表示，并用Wilcoxon检验或Kruskal-Wallis检验进行比较，计数资料以频数或百分比表示，采用卡方检验或Fisher确切概率法进行比较。采用多因素Logistic回归分析手术影响因素。P<0.05为差异有统计学意义。

## 2 结果

### 2.1 病例临床特征

本研究纳入的385例病例分为三组，包括楔形切除组155例，肺段切除组175例，肺叶切除组55例。共有270例（70.13%）女性和115例（29.87%）男性，平均年龄（52.88±12.90）岁。总体手术部位分布：右上肺123例（31.95%），右下肺73例（18.96%），左上肺119例（30.91%），左下肺70例（18.18%）。术中中位出血量为71.00（54.00, 88.00）mL，手术中位时长85.00（55.50, 118.00）min，住院中位时长为6.00（5.00, 7.00）d。术后发生持续性漏气（>5 d）的有17例（4.42%）。术后病理类型主要为微浸润型腺癌151例，其次为浸润性腺癌135例，原位腺癌86例，鳞癌7例，腺鳞癌3例，典型类癌3例。术中切除的淋巴结中位组数为2（0, 4）组，未出现术后淋巴结转移阳性病例（表1）。其中肺段组是采用“以病灶为中心，肺亚段为解剖单元”的手术策略，具体手术分布见表2。肺段组切除的中位亚段数为3（3, 4）个。

**表1:** 病例临床资料

Variable	Total (n=385)	Lobectomy (n=55)	Segmentectomy (n=175)	Wedge resection (n=155)	F/H/χ²	P
Age (yr), Mean±SD	52.88±12.90	57.47±10.77	53.93±11.84	50.06±14.12	8.038	<0.001
Gender, n (%)					3.570	0.168
Female	270 (70.13)	37 (67.27)	116 (66.29)	117 (75.48)		
Male	115 (29.87)	18 (32.73)	59 (33.71)	38 (24.52)		
Intraoperative blood loss (mL), Md (Q₁, Q₃)	71.00 (54.00, 88.00)	83.00 (72.00, 91.00)	84.00 (73.00, 98.00)	51.00 (44.00, 57.00)	226.509	<0.001
Operative duration (min), Md (Q₁, Q₃)	85.00 (55.50, 118.00)	102.00 (85.00, 120.00)	110.00 (90.00, 136.00)	53.00 (44.00, 64.00)	221.576	<0.001
Length of hospital stay (d), Md (Q₁, Q₃)	6.00 (5.00, 7.00)	7.00 (6.00, 9.00)	7.00 (6.00, 8.00)	5.00 (5.00, 6.00)	81.321	<0.001
Air leakage after pulmonary resection (>5 d), n (%)					2.520	0.284
No	368 (95.58)	51 (92.73)	166 (94.86)	151 (97.42)		
Yes	17 (4.42)	4 (7.27)	9 (5.14)	4 (2.58)		
Histologic type, n (%)					70.936	<0.001
Adenocarcinoma in situ	86 (22.34)	3 (5.45)	30 (17.14)	53 (34.19)		
Minimally invasive adenocarcinoma	151 (39.22)	9 (16.36)	68 (38.86)	74 (47.74)		
Invasive adenocarcinoma	135 (35.06)	39 (70.91)	72 (41.14)	24 (15.48)		
Other	13 (3.38)	4 (7.27)	5 (2.86)	4 (2.58)		
Number of lymph node stations, Md (Q₁, Q₃)	2 (0, 4)	5 (4, 6)	3.00 (1, 4)	0 (0, 1)	179.844	<0.001
Lymph node metastasis, n	0	0	0	0		

**表2:** 肺段组手术分布

Right upper lobe	n	Right lower lobe	n	Left upper lobe	n	Left lower lobe	n
S^1^	5	S^6^	9	S^1+2^	7	S^6^	10
S^1^+S^2^	4	S^6^+S^*^	1	S^1+2^+S^3^	12	S^6^+S^8^a	2
S^1^+S^2^a	2	S^6^+S^8^a	1	S^1+2^+S^3^a	4	S^6^+S^8^a+S^9^a	1
S^1^a+S^2^	2	S^6^+S^10^a	2	S^1+2^+S^3^c	1	S^6^b+S^8^	1
S^1^a+S^2^a	1	S^6^+S^10^c	1	S^1+2^a+b	5	S^6^b+S^8^a+S^9^a	4
S^1^+S^3^	1	S^6^+S^9^a+S^10^a	2	S^1+2^a+b+S^3^a	1	S^8^	2
S^2^	12	S^6^b+S^8^a	1	S^1+2^b+c	1	S^8^a+S^9^a	1
S^2^+S^3^a	2	S^6^b+S^8^a+S^9^a	2	S^1+2^c+S^3^a+S^4^a	2	S^10^	4
S^2^b+S^3^a	2	S^6^c	1	S^1+2^c+S^3^a	2	S^8^+S^9^a	1
S^3^	6	S^7^+S^8^	1	S^1+2^c+S^3^a+S^4^+S^5^	2	S^8^+S^9^	3
S^3^b	2	S^7^a+S^8^	1	S^1+2^c+S^4^a	1	S^8^+S^9^b	2
S^1^b+S^3^	4	S^8^	2	S^3^a+b	1		
S^1^b+S^3^b	1	S^9^+S^10^	2	S^3^b+c	3		
S^1^bii+S^3^	1	S^9^+S^8^b	1	S^3^b+S^4^b	1		
S^3^bⅱ	1	S^6^a+b+S^*^+S^8^a	1	S^4+5^	11		
		S^6^b+S^7^b	1	S^4^a	1		
		S^8^+S^9^a	1	S^1+2^a+b+S^3^c	1		
		S^10^	2	S^1+2^a+S^3^b+c	1		
				S^1+2^a+S^3^c	2		
				S^3^	7		

The median number of subsegments resected in segmentectomy is 3 (3, 4).

### 2.2 三组深度比较

本研究基于肺段解剖结构，采用结节所属的肺段开口至结节内缘的距离（OA值）和结节内缘至壁层胸膜的距离（AB值）作为结节位置深度指标（图1）。OB为结节所在肺段开口经过结节中心至胸膜的距离，肺叶组、肺段组和楔形组的OB值分别为7.21（6.36, 8.02）cm、7.41（6.72, 8.23）cm和7.63（6.92, 8.41）cm，三组间并无统计学差异（P=0.113）。肺叶组结节中位深度（OA值）为3.04（1.80, 4.18）cm，肺段组和楔形组分别为4.43（3.27, 5.43）cm、6.05（5.26, 6.85）cm。以AB作为指标，肺叶组结节中位深度为3.57（2.60, 4.85）cm，肺段组为3.01（2.26, 4.03）cm，楔形组为1.53（1.16, 1.97）cm。三组之间OA值与AB值均存在显著差异（P<0.001）（图2）。

**图1:**
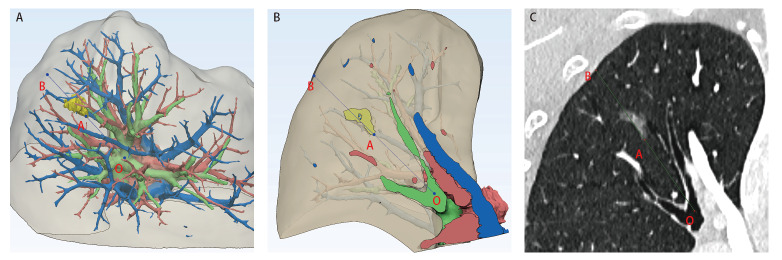
右上肺结节。A：肺结节三维示意图；B：三维深度示意图；C：二维深度测量图。O点为肺段开口，A点、B点分别是肺段开口至结节中心的连线与结节内缘和壁层胸膜的交点。

**图2:**
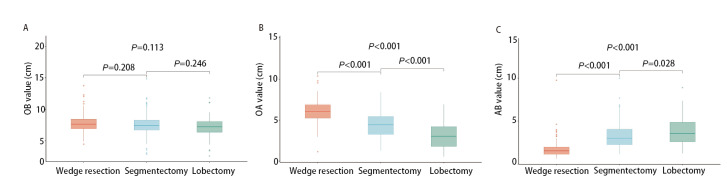
楔形组、肺段组与肺叶组结节深度比较。A：三组OB值无统计学差异；B：三组OA值逐渐减小；C：三组AB值逐渐增大。

### 2.3 肺段组与楔形组对比

肺段切除组和楔形切除组在实性成分占比（consolidation-to-tumor ratio, CTR）、手术部位分布没有统计学差异。肺段组结节最大径平均为（1.31±0.34）cm，楔形组则是（1.08±0.29）cm（P<0.001）。在结节的深度（AB值）对比上，楔形组比肺段组更短（P<0.001），表明位置更浅。肺段组2 cm切缘球涉及中位亚段数为2（2, 3）个，多于楔形组的1（1, 2）个（P<0.001）。肺段组中，有140例（80.00%）的结节内缘至胸膜的距离超过2 cm，而楔形组中仅有35例（22.58%）（表3）。在多因素Logistic回归分析中，结节最大径（P=0.010）、OA值（P<0.001）、AB值>2（P<0.001）、亚段数（P<0.001）均显示出统计学差异（表4）。

**表3:** 肺叶组、肺段组和楔形组手术相关特征

Variable	Lobectomy (n=55)	Segmentectomy (n=175)	Wedge resection (n=155)	Lobectomy vs Segmentectomy			Segmentectomy vs Wedge resection	
				t/Z/χ²	P		t/Z/χ²	P
Tumor size (cm), Mean±SD	1.50±0.35	1.31±0.34	1.08±0.29	3.543	<0.001		6.813	<0.001
Consolidation tumor ratio, n (%)				5.950	0.015		2.091	0.148
≤0.5	34 (61.82)	137 (78.29)	131 (84.52)					
>0.5	21 (38.18)	38 (21.71)	24 (15.48)					
Tumor location lobe, n (%)				12.045	0.007		4.053	0.256
Right upper	23 (41.82)	46 (26.29)	54 (34.84)					
Right lower	15 (27.27)	32 (18.29)	26 (16.77)					
Left upper	8 (14.55)	66 (37.71)	45 (29.03)					
Left lower	9 (16.36)	31 (17.71)	30 (19.35)					
Forced vital capacity (L)	2.74 (2.20, 3.10)	2.89 (2.50, 3.40)	2.90 (2.50, 3.50)	-1.696	0.090		-0.087	0.931
Forced expiratory volume in 1 s (L)	2.32 (2.0, 2.8)	2.53 (2.10, 2.90)	2.48 (2.20, 2.90)	-1.528	0.127		-0.378	0.706
OA value (cm), Md (Q₁, Q₃)	3.04 (1.80, 4.18)	4.43 (3.27, 5.43)	6.05 (5.26, 6.85)	-4.821	<0.001		-10.026	<0.001
Subsegments^a^, Md (Q₁, Q₃)	5.00 (4.00, 6.00)	2.00 (2.00, 3.00)	1.00 (1.00, 2.00)	-9.481	<0.001		-10.822	<0.001
AB value (cm)								
Md (Q₁, Q₃)	3.57 (2.60, 4.85)	3.01 (2.26, 4.03)	1.53 (1.16, 1.97)	-2.199	0.028		-11.471	<0.001
>2, n (%)	47 (85.45)	140 (80.00)	35 (22.58)	0.819	0.365		108.800	<0.001
≤2, n (%)	8 (14.55)	35 (20.00)	120 (77.42)					
Margin distance (cm), Md (Q₁, Q₃)	3.00 (2.00, 4.00)	2.50 (1.50, 3.00)	1.50 (1.15, 2.00)	-1.825	0.068		-6.962	<0.001
Margin distance/Tumor size, n (%)				0.068	0.794		11.422	<0.001
≥1	47 (85.45)	155 (88.57)	115 (74.19)					
<1	8 (14.55)	20 (11.43)	40 (25.81)					

^a^Number of subsegments involved within a 2 cm margin sphere.

**表4:** 肺段组和楔形组多因素Logistic回归分析

Variable	Beta	SE	Wald	P	OR	95%CI
Age	0.002	0.014	0.013	0.909	1.00	0.97-1.03
Tumor size	-1.488	0.581	6.555	0.010	0.23	0.07-0.71
OA value	0.473	0.131	13.034	<0.001	1.61	1.24-2.08
AB value>2	1.919	0.340	31.911	<0.001	6.81	3.50-13.26
Subsegments^a^	-1.400	0.258	29.397	<0.001	0.25	0.15-0.41

^a^Number of subsegments involved within a 2 cm margin sphere.

### 2.4 肺叶组与肺段组对比

肺叶组结节平均最大径为（1.50±0.35）cm，比肺段组更大（P<0.001），位置也更深（P<0.001）。三维重建中2 cm切缘球所涉及的肺亚段数肺叶组中位数量是5（4, 6）个，显著多于肺段组的2（2, 3）个（P<0.001），也显著多于肺段组中实际手术切除的3个的中位亚段数（P<0.001）。两组病例在CTR（P=0.015）、手术部位分布（P=0.007）上也存在差异，肺叶组较肺段组CTR>0.5的比例更高（38.18% vs 21.71%）。肺段组中，左肺上叶结节占比最高（37.71%），高于肺叶组的左上肺结节占比（14.55%）（表3）。多因素Logistic回归分析结果显示，2 cm切缘球涉及的亚段数量是决定采用肺叶切除还是肺段切除的重要影响因素（P<0.001, OR=0.14, 95%CI: 0.07-0.26）。此外，不同的手术部位对手术方式的影响有统计学差异，这提示肺叶本身的结构也是影响因素之一（表5）。

**表5:** 肺叶组和肺段组多因素Logistic回归分析

Variable	Beta	SE	Wald	P	OR	95%CI
Age	0.001	0.024	0.001	0.981	1.00	0.95-1.05
Tumor size	-1.017	0.796	1.633	0.201	0.36	0.08-1.72
Consolidation tumor ratio	0.790	0.562	1.975	0.160	2.20	0.73-6.62
Tumor location lobe						
Right upper	-3.223	0.791	16.628	<0.001	0.04	0.01-0.19
Right lower	-1.708	0.792	4.653	0.031	0.18	0.04-0.86
Left upper			17.026	0.001		
Left lower	-1.544	0.852	3.282	0.070	0.21	0.04-1.14
OA value	-0.181	0.234	0.598	0.439	0.84	0.53-1.32
AB value	0.211	0.232	0.827	0.363	1.24	0.78-1.95
Subsegments^a^	-1.991	0.323	37.921	<0.001	0.14	0.07-0.26

^a^Number of subsegments involved within a 2 cm margin sphere.

### 2.5 亚肺叶手术切缘比较

肺段切除获得的中位切缘宽度为2.50（1.50, 3.00）cm，明显大于楔形切除的1.50（1.15, 2.00）cm（P<0.001）。若以结节直径大小作为切缘宽度的标准，肺段切缘依旧优于楔形切缘[切缘/直径≥1，155（88.57%） vs 115（74.19%）（P<0.001）]（表3）。进一步分析楔形组中，当切缘不足2 cm时，结节内缘至胸膜距离超过2 cm的病例占29.03%，高于切缘≥2 cm的12.90%（P=0.019）。使用结节大小作为切缘标准时，切缘/直径<1的病例中，结节内缘至胸膜距离>2 cm的比例为37.50%，切缘/直径≥1中的占比为17.39%，两者存在统计学差异（P=0.009）（表6）。

**表6:** 楔形组中不同切缘标准的比较

Variable	Margin distance					Margin distance/Tumor size			
	<2 cm (n=93)	≥2 cm (n=62)	t/χ²	P		<1 (n=40)	≥1 (n=115)	t/χ²	P
Age (yr), Mean±SD	48.46±14.52	52.47±13.25	-1.742	0.084		53.13±13.57	49.00±14.21	1.600	0.112
Tumor size (cm), Mean±SD	1.06±0.28	1.11±0.30	-0.963	0.338		1.22±0.33	1.03±0.26	3.456	0.001
Tumor location lobe, n (%)			4.253	0.235				1.123	0.771
Right upper	29 (31.18)	25 (40.32)				13 (32.50)	41 (35.65)		
Right lower	13 (13.98)	12 (19.35)				7 (17.50)	18 (15.65)		
Left upper	28 (30.11)	17 (27.42)				10 (25.00)	35 (30.43)		
Left lower	23 (24.73)	8 (12.90)				10 (25.00)	21 (18.26)		
AB value, n (%)			5.536	0.019				6.864	0.009
≤2	66 (70.97)	54 (87.10)				25 (62.50)	95 (82.61)		
>2	27 (29.03)	8 (12.90)				15 (37.50)	20 (17.39)		

## 3 讨论

JCOG0802和CALGB140503研究^[3,4]^结果表明，亚肺叶切除治疗直径≤2 cm的外周早期肺癌肿瘤学疗效不亚于甚至优于肺叶切除术。中华医学会肺癌诊疗指南^[12]^推荐肺段切除可治疗含磨玻璃成分的直径≤2 cm的外周早期肺癌。亚肺叶切除的安全切缘是良好肿瘤学疗效的重要前提^[13⇓-15]^，必须满足切缘宽度≥2 cm或肿瘤直径^[16]^。本研究纳入病例绝大部分为含磨玻璃成分的早期肺癌，大小均符合亚肺叶切除指征，术前按照切缘要求设计手术，包括楔形切除155例，肺段切除175例，肺叶切除55例，三种手术方式的肺组织切除范围依次增大。

楔形、肺段和肺叶切除组的肺结节大小逐渐增大，分别为（1.08±0.29）cm、（1.31±0.34）cm和（1.50±0.35）cm（P<0.001），说明大小是影响手术方式的因素之一。除了大小以外本研究还分析了肺结节深度对手术方式的影响。目前对于肺结节深度的分类为外周和深部，外周结节为肺结节中心位于肺实质外周1/3，此外即为深部，该分类用比例值衡量，但测量标准不统一^[17,18]^。本研究采用深度绝对值测量，将OA和AB作为结节的深度位置指标。楔形、肺段和肺叶切除组的OA值分别为6.05（5.26, 6.85）cm、4.43（3.27, 5.43）cm和3.04（1.80, 4.18）cm（P<0.001），AB值分别1.53（1.16, 1.97）cm、3.01（2.26, 4.03）cm和3.57（2.60, 4.85）cm（P<0.001），说明肺结节深度也是手术方式的影响因素。本研究首次用数据证实越大越深的肺结节，需要切除更大范围的肺组织才能获得安全切缘。

在亚肺叶切除中楔形和肺段切除切缘宽度有明显差异。尽管楔形组的肺结节较肺段组更小更浅，但肺段切除获得的中位切缘宽度为2.50（1.50, 3.00）cm，显著大于楔形切除的1.50（1.15, 2.00）cm（P<0.001），说明肺段较楔形具有切缘优势，其本质是通过解剖性切除更大的肺组织获得安全切缘。JCOG0804研究^[1]^显示，原计划实施楔形切除333例CTR≤0.25、肿瘤大小≤2 cm的早期肺癌，最终结果为楔形264例，肺段58例，肺叶11例，结果分析认为深部肺结节楔形切除难以获得安全切缘。在满足亚肺叶切除指征的前提下，楔形和肺段切除该如何选择，目前标准尚不明确。本研究显示，当楔形切除切缘<2 cm时，AB值>2 cm的病例占29.03% ，而切缘≥2 cm时，AB值>2 cm的病例为12.90%（P=0.019）。此结果提示当结节的内缘至胸膜的距离超过2 cm时，楔形切除的手术方式可能并不是最佳选择，而应该选择肺段切除术以确保安全切缘。AB值兼具有肺结节大小和深度信息，较其他指标如结节距离胸膜的最小距离^[19]^更能符合切缘要求。

肺叶和肺段两组单因素分析提示结节最大径、肺段组和肺叶组在CTR（P=0.015）、手术部位（P=0.007）有统计学差异。多因素Logistic回归分析表明影响肺叶切除与肺段切除选择的主要因素仅为2 cm切缘涉及的亚段数和结节所在的肺叶。本研究肺叶组结节如行肺段切除术所累涉及的中位亚段数为5个，显著多于肺段组3个（P<0.001）。Nomori等^[10]^报道，当肺段切除需切除≥5个亚段时，肺段切除对肺功能的保护与肺叶切除无显著差异。也有研究^[11]^指出，当肺段切除亚段数超过肺叶总亚段数一半时，无保护肺功能的效果。因此，术前3D-CTBA重建模拟手术规划切缘和切除范围可有效避免范围过大的肺段切除^[20,21]^。本研究还显示左上肺结节肺段切除的比例（37.71%）高于右上肺（26.29%）。左上肺的解剖结构等同于右上叶和右中叶，更多的亚段数提供了更多肺段切除的可能性。相比于上肺叶，下肺叶各肺段间的夹角更小，不仅深部结节的2 cm切缘球涉及的亚段数会更多，而且肺段切除更难获得足够的手术切缘宽度。

本文首次用数据证实肺结节大小深度对肺切除手术方式影响的规律性，但除此规律性以外，还存在主观选择性。在实际工作中术者对于以实性成分为主的早期肺癌，在肺段和肺叶切除的选择中，可能更倾向于肺叶切除，所以肺结节CTR值肺叶组明显大于肺段组。

本研究为单中心回顾性研究，纳入研究的病例数较少。随访时间尚短，还需进一步跟踪随访以评估远期疗效。对于楔形切除与肺段切除的选择指征还需更多的前瞻性研究进一步明确。

综上，肺结节的大小、深度综合影响楔形、肺段和肺叶切除手术方式的选择，越深越大的肺结节需要切除更大范围的肺组织才能获得安全切缘。肺结节内缘距离最近胸膜≤2 cm可能是楔形切除的理想指征，位置更深的结节需术前充分评估肺段切除的肿瘤学疗效与肺功能保留效果以决定是否采取肺叶切除手术方式。
